# Preparation and Evaluation of Caffeine Orodispersible Films: The Influence of Hydrotropic Substances and Film-Forming Agent Concentration on Film Properties

**DOI:** 10.3390/polym15092034

**Published:** 2023-04-25

**Authors:** Robert-Alexandru Vlad, Andrada Pintea, Mădălina Coaicea, Paula Antonoaea, Emőke Margit Rédai, Nicoleta Todoran, Adriana Ciurba

**Affiliations:** 1 Pharmaceutical Technology and Cosmetology Department, Faculty of Pharmacy, George Emil Palade University of Medicine, Pharmacy, Science and Technology of Targu Mures, 38th Gheorghe Marinescu Street, 540142 Targu Mures, Romania; 2 Targu Mures Clinical County Hospital, 6th Bernady Gyorgy Street, 540072 Targu Mures, Romania; 3 Catena Hygeia Darmanesti, 1st Muncii Street, 605300 Bacau, Romania

**Keywords:** caffeine, orodispersible films, hydrotropic effect, pharmacotechnical evaluation, dissolution

## Abstract

This study aimed to develop caffeine (CAF) orodispersible films (ODFs) and verify the effects of different percentages of film-forming agent and hydrotropic substances (citric acid—CA or sodium benzoate—SB) on various film properties. Hydroxypropyl methylcellulose E 5 (HPMC E 5) orodispersible films were prepared using the solvent casting method. Four CAF-ODF formulations were prepared and coded as CAF1 (8% HPMC E 5, CAF), CAF2 (8% HPMC E 5 and CAF:CA–1:1), CAF3 (9% HPMC E 5 and CAF:CA–1:1), and CAF4 (9% HPMC E 5 and CAF:SB–1:1). The CAF-ODFs were evaluated in terms of disintegration time, folding endurance, thickness, uniformity of mass, CAF content, thickness-normalized tensile strength, adhesiveness, dissolution, and pH. Thin, opaque, and slightly white CAF-ODFs were obtained. All the formulations developed exhibited disintegration times less than 3 min. The dissolution test revealed that CAF1, CAF2, and CAF3 exhibited concentrations of active pharmaceutical ingredients (APIs) released at 30 min that were close to 100%, whilst CAF4 showed a faster dissolution behaviour (100% of the CAF was released at 5 min). Thin polymeric films containing 10 mg of CAF/surface area (3.14 cm^2^) were prepared.

## 1. Introduction

One of the most common issues concerning pediatric and geriatric patients is difficulty swallowing, which prevents the administration of a drug in solid dosage forms. Therefore, a new dosage form has been developed to quickly and efficiently provide drugs via the oral route without water intake. Orodispersible films (ODFs) are an attractive delivery system, with increased patient compliance, providing drugs at the site of action with prompt disintegration, dissolution, and administration without the need for swallowing and chewing [1,2,3].

ODFs are single-layer or multi-layer thin polymer sheets intended for rapid dissolution or disintegration in the oral cavity. They are usually applied directly to the tongue [4]. An ideal ODF should be thin and flexible, but robust to mechanical forces. Additionally, the film should not be sticky and should hold its form without rolling [5]. The main methods used to manufacture orodispersible films are the solvent casting method, hot melt extrusion, electrostatic spinning, inkjet, and flexographic printing [6,7].

**Figure 1:**
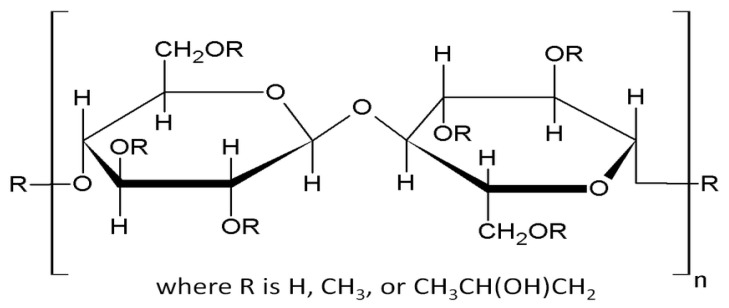
The chemical structure of HPMC.

Various polymers, such as hydroxypropyl cellulose (HPC), polyvinylpyrrolidone (PVP), polylactic acid (PLA), polyvinyl alcohol (PVA), and hydroxypropyl methylcellulose (hypromellose, HPMC), can be used for the preparation of ODFs [8]. HPMC is a swellable, water-soluble polymer that enhances the sustained-release proprieties of active ingredients in pharmaceuticals and is used for immediate-release dosage forms [9]. It is widely implemented in the pharmaceutical manufacturing industry as a binder, thickening agent, hydrophilic matrix material, and film-forming material [10]. Hydroxypropyl methylcellulose is a partly O-methylated O-(2-hydroxypropylated) cellulose (Figure 1), and it is classified by several factors, such as viscosity and the degree of substitution; HPMC E 5, HPMC E 6, and HPMC E 15 are low-viscosity polymers [8,11]. Active pharmaceutical ingredients (APIs), such as furosemide, fenofibrate, naproxen, aripiprazole, mirtazapine, or antimicrobial agents, can be incorporated in a hydroxypropyl methylcellulose ODF [8,12,13,14,15].

**Figure 2:**
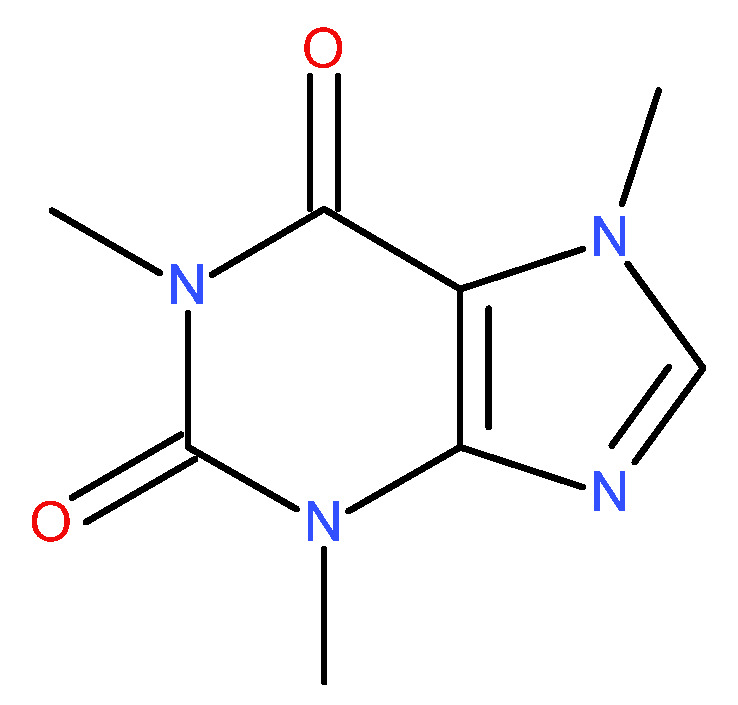
The chemical structure of CAF.

Caffeine (CAF) is a methylxanthine alkaloid (Figure 2) found in different parts of plants, such as beans, nuts, seeds, and leaves. When in a pure form, it is an odorless white solid powder that is soluble in boiling water and in organic solvents, such as ethanol, acetone, chloroform, and benzene [16,17]. Additionally, it is rapidly and completely absorbed while ingested, with 95% being absorbed within 45 min after its intake. Moreover, it has a high liposolubility which enables it to pass through all biological membranes and cross the blood-brain barrier [18,19]. It is almost exclusively metabolized in the liver by the cytochrome P450 enzyme system to paraxanthine, theobromine, and theophylline, with no first-pass metabolism [20,21,22]. It is structurally similar to adenosine, an endogenous neuromodulator, and has three underlying mechanisms of action. CAF is a naturally occurring central nervous system excitant, and it can be used as a psychoactive stimulant. The main and most important one is the antagonism at the level of adenosine receptors in the central nervous system, which modulates the release of different neurotransmitters, such as glutamate, dopamine, serotonin, and noradrenaline [23,24]. Its effects due to phosphodiesterase inhibition and intracellular calcium mobilization require much higher concentrations of active drugs and therefore are not as noticeable [25,26,27].

This alkaloid is the most widely consumed psychoactive drug. It enhances attention and vigilance, stabilizes mood, and promotes wakefulness. Furthermore, studies have shown that this substance can improve cognitive performance, enabling learning and memory in tasks by maintaining one’s intellectual activity, resulting in positive effects on both long-term and short-term memory in both human and animal models [28,29,30]. Other effects of CAF include decreased sleep and muscle strength in human models [31]. It is used as an antidepressant, in the treatment of apnea in premature neonates, and to improve concentration [32,33].

ODFs mainly consist of a polymer with a film-forming capacity that serves as an active drug ingredient or drug carrier. One advantage is that the polymer can be broken down rapidly by the saliva and disintegrate in a few seconds, allowing the drug to be absorbed through the oral cavity, which delivers it into systemic circulation. Due to the high vascularization and thin-membrane structure of the sublingual mucosa, it offers very good bioavailability [2,34]. These new systems of drug transportation have become more and more popular in the scientific literature. Statistics have shown that four out of five patients prefer orally disintegrating dosage forms over conventional solid oral dosage forms [35]. ODFs are manufactured to contain a precise drug load limited to 50 mg per ODF, which allows only medium-potency substances to be included [36].

However, there is a lack of research studies exploring ODF characteristics with a potential impact on end-user acceptability [37]. In addition to that, the shortage of medicines designed to be used for pediatric practice is a pressing problem that often requires the extemporaneous manipulation of dosage forms for adults [38]. Different APIs have been selected as model ingredients in studies that aimed to develop ODFs, such as rupatadine [1], prednisolone [6], mirtazapine [8], and aripiprazole [12].

The objective of this study is to develop new formulations of CAF-ODFs that fulfill the required characteristics and to analyze their properties using hydrotropic ingredients (citric acid—CA and sodium benzoate—SB), sweeteners (sucralose), solvents (distilled water), and co-solvents (ethanol), because these types of pharmaceutical formulations are produced by only a few companies: CAF (Nanoveda New Delhi, India), Aavishkar (Hyderabad, India), BonAyu Life Sciences (Uxbridge, UK), and Revvies^®^ Energy strips (Engadine, Australia). All of these formulations are registered as dietary supplements. As can be seen in Table 1, the dietary supplements that comprise CAF-ODFs are Revvies^®^ Energy strips, Nanoveda, and BonAyu, whilst Aavsishkar does not include this information [39,40,41,42]. Other studies that are mentioned in Table 1 have had the same aim of developing CAF-ODFs. In the study conducted by Draskovic et al., a combination of polymers (PVA, HPC) and a single plasticizer was used (polyethylene glycol (PEG)) [43]. Sultana et al., presented three different polymers that could be used to develop CAF-ODFs, with one of the selected film-forming agents being HPMC-2910 (15 cPs); however, in this case, HPMC with a higher viscosity was employed in comparison to our study, in which HPMC E 5 is used (5 cPs) [44]. Other film-forming agents that have been used to develop CAF-ODFs include the following: sodium alginate + sodium starch glycolate; Kollicoat^®^ IR white (BASF Pharma, Florham Park, NJ, USA); and Blanose Carboxymethyl Cellulose Type 7HF-PH (Table 1) [3,43,44,45].

## 2. Materials and Methods

### 2.1. Preparation of ODFs

For the preparation of CAF-ODFs, the following ingredients were used: Vivapharm^®^ HPMC E 5 (average content of methoxyl groups is 29% and the hydroxypropyl groups is 10%; viscosity of 2% dispersion is 5 cPs) as the film-forming agent (JRS PHARMA, Rosenberg, Germany), 1,2-propylene glycol (99.7%) as the plasticizer (Scharlau, Barcelona, Spain), sucralose as the sweetener (99%) (Myprotein, Bucharest, Romania), CAF (98.5%) (Mayam, Bucharest, Romania), CA as the hydrotropic substance (99.5%) (ViVoCHem, Almelo, The Netherlands), SB as the hydrotropic substance (99%) (Gazdabolt, Budapest, Hungary), 96% ethanol (*v*/*v*) as the co-solvent (Chimreactiv S.R.L, Romania), and ultrapure water as the solvent (Direct Q3 System, Millipore, Bucharest, Romania).

In the present study, the solvent casting method was used to prepare the orodispersible films. Four formulations (coded CAF1-4) were prepared, as presented in Table 2. CAF, HPMC E 5 (film-forming agent), and sucralose (sweetener) were added into distilled water (solvent) and dispersed at room temperature. 1,2-propylene glycol (plasticizer) and ethylic ethanol (co-solvent) were then slowly added and stirred using an MS-H280-Pro DLAB agitator (DLAB, La Mirada, CA, USA) for 30 min at 1000 rotations/min at room temperature. A 9.5 g dispersion was poured into a Petri glass with a total surface area of 86.54 cm^2^ and kept at a controlled room temperature (20 ± 2 °C) for 24 h. For the second, third, and fourth film formulations, the same process was repeated, with the difference in that the first step of preparation consisted in kneading the CA or SB with the pre-established amount of caffeine (2.88 g each). For CAF2 and CAF3, CA was used as a hydrotropic substance, whilst for CAF4, SB was used as a hydrotropic substance. Besides being a hydrotropic substance, CA is a sialagogue ingredient, increasing one’s saliva flow; moreover, it can also act as a plasticizer, increasing film plasticity. For further evaluations, the prepared ODFs were manually cut, employing a sharpened circular mold with a diameter of 2 cm (surface area: 3.14 cm^2^). When SB and caffeine were used as a mixture to develop CAF-ODFs with a concentration of 8% HPMC E 5, the ODFs developed presented a high adhesivity so they were eliminated from the study.

### 2.2. pH

To evaluate the pH, one film (total surface area of 3.14 cm^2^) from each formulation was dispersed in 20 mL of ultrapure water. Subsequently, the solution was filtered and the pH of the filtrate was measured using a pH meter (pH Check, TFA Dostmann, Wertheim am Main, Germany) [46,47]. The experiment was conducted in triplicate, and the results were expressed as the average ± standard deviation (SD).

### 2.3. CAF-ODFs’ Uniformity of Mass and Thickness

The uniformity of mass was determined with the help of a KERN scale (Berlin, Germany), and the results are expressed as the average weight ± SD. To determine the thickness of the films, 10 ODFs were measured in five different spots using a digital micrometer with a precision of 0.01 µm (Yuzuki, Mumbai, India) [48].

### 2.4. Mechanical Properties

The folding endurance was determined by folding the film manually at the same central line that divided the film (shaped as a disc with a surface area of 3.14 cm^2^) into two equal parts until it broke [49]. This test was repeated for five films for each formulation, and the average folding endurance ± SD was calculated.

Tensile strength is the ability of plastic material to withstand a maximal amount of tensile stress without failure. The stress occurs while the material is being pulled or stretched. Since in this study, the thickness is considered whilst calculating the “tensile strength”, this parameter will be further referred to as thickness-normalized tensile strength.

By testing the thickness-normalized tensile strength, the ability of the film to resist breaking was evaluated. This parameter was assessed using an in-house manufactured instrument. The evaluated CAF-ODFs were placed between two clamps (the superior clamp was fixed on a support whilst the inferior one was mobile). On the inferior part, weights of 5 g were successively attached until the film broke [46]. Five determinations for each CAF-ODF formulation were conducted using ODFs shaped as discs with a total surface area of 3.14 cm^2^, and the final mass at which the film broke was considered as M. To calculate the thickness-normalized tensile strength, Equation (1) was applied.

TS (N·mm^−2^) = (M × g)/(W × T) (1)

where

TS—thickness-normalized tensile strength (N·mm^−2^);
M—the total weight at which the sample cracked/broke;
g—(constant) gravitational acceleration (9.81 N·kg^−1^);
W—sample width (mm);
T—sample average thickness measured in five spots (mm).

### 2.5. Disintegration Behavior

The disintegration behavior was evaluated through two methods: The slide frame method (met1) and modified pharmacopoeia method (met2).

#### 2.5.1. Method 1: Slide Frame Method

The films (area: 3.14 cm^2^, *n* = 6) were fixed in a slide frame which was placed on a beaker, and three drops of water were placed on top of the film surface area using an Eppendorf pipette (37 ± 2 °C) [50,51]. The average time needed for the ODFs to disintegrate was calculated considering the time required for the water to dissolve the film matrix. The endpoint was considered as the first drop of water falling on the ground of the beaker.

#### 2.5.2. Method 2 (met2)

For this test, a basket-rack assembly was employed in a Biobase TFUT-3 tester (Tablet Four-Usage Tester, Biobase, Jinan, China). Six films were separately placed in the six tubes of the basket-rack assembly, and this assembly was positioned inside a glass beaker containing 600 mL of distilled water at 37 ± 2 °C. The evaluated samples were considered to have disintegrated either when there was no sample residue left in the tube at the end of the assay, or when a soft mass with no palpably firm core was present [51].

### 2.6. Adhesivity Test

The adhesivity test was performed using a modified Chirana scale. The disc-shaped film (area: 3.14 cm^2^) was placed between the left plate of the scale and a metallic surface. Two drops of water were added to moisten the sample. On the left plate, a small weight of two grams was added to apply just a small pressure. On the right plate, weights in ascending order were added until the left plate was detached from the film. The adhesivity was considered as the vertical tensile force determined by the mass that caused the detachment of the plate from the metallic surface on which the CAF-ODF was placed. The detachment force was expressed in dynes/cm^2^ and was calculated using Equation (2):

F = m × g/A,(2)

where

m—the applied mass needed for detachment (kg);
g—(constant) gravitational acceleration (9.81 N·kg^−1^);
A—ODFs’ film surface area: 3.14 cm^2^.

### 2.7. CAF Content

To assay the amount of drug for each formulation, a film was dissolved in a 5 mL phosphate buffer solution (pH = 6.8), which consisted of 28.20 g of disodium hydrogen phosphate (Fisher Scientific, Bucharest, Romania), 11.45 g of potassium dihydrogen phosphate (Chimopar, Bucharest, Romania), and adjusted with water to 1000 mL. A dilution was performed for each film and the final solution was analyzed using the UV-1800 Shimadzu Spectrophotometer (Mettler Toledo, Columbus, OH, USA) against a blank solution. The absorbance was read for each sample at the specific wavelength of 273 nm using a previously prepared calibration curve with the following concentrations: 1 µg/mL, 2.5 µg/mL, 5 µg/mL, 10 µg/mL, 15 µg/mL, and 20 µg/mL.

### 2.8. Dissolution Test

The Erweka DT light Series (Erweka GmbH, Langen (Hessen), Germany) with a basket was used because it prevents the film from floating, and it eases the process of assessing the API. The films were added to the basket and phosphate buffer (pH = 6.8) solution with the same composition as described in Section 2.7. A volume of 900 mL was added and kept at 37 ± 0.5 °C. Every 3, 5, 10, 15, 20, and 30 min, 5 mL aliquot was withdrawn and replaced with the same quantity of phosphate buffer solution (maintained at the same temperature of 37 ± 0.5 °C). The samples were analyzed using UV-1800 Shimadzu Spectrophotometer (Mettler Toledo, Columbus, OH, USA). The % of CAF released was evaluated using the spectrophotometric method previously described in Section 2.7 [2,52].

### 2.9. Statistical Evaluation

For all the parameters described below, a statistical evaluation was performed with the help of GraphPad Prism 9 software (Dotmatics, Boston, MA, USA) by means of the Brown-Forsythe ANOVA and Welch one-way ANOVA tests. The results are represented as average ± SD. The significance level was set to 0.05 (*p*) with the *p* values presented as asterisks in the results and discussion sections:

- ns (*p* > 0.05), ns—not significant;
* (*p* ≤ 0.05);
** (*p* ≤ 0.01);
*** (*p* ≤ 0.001);
**** (*p* ≤ 0.0001).

## 3. Results and Discussion

New CAF-ODF formulations are described in this article in which different concentrations of film-forming agents and hydrotropic substances (CA or SB) are selected. Even though HPMC is a polymer extensively used to develop ODFs, until now this polymer with a viscosity of 5 cPs had not been selected as a film-forming agent for CAF-ODFs. Additionally, the presence of SB in the composition as a hydrotropic substance is a novelty for this type of pharmaceutical formulation. The CAF-ODFs were evaluated in terms of disintegration time, folding endurance, thickness, uniformity of mass, CAF content, thickness-normalized tensile strength, adhesiveness, dissolution, and pH, considering current pharmacopeial requirements or the pre-established methods previously published. All of the results are described in the sections below.

The liquid that was selected to evaluate the disintegration behavior for the methods detailed below was water, because its pH is close to the pH of saliva. Even though this solvent is used intensively to evaluate the disintegration time, some limitations need to be considered. For example, saliva presents a higher ionic strength in comparison to that of water. Additionally, their viscosities are different: the viscosity of saliva tends to be higher in comparison with the viscosity of water. Moreover, the composition and the buffering capacity of both saliva and water are two parameters that differentiate them, a fact that might imply they have different disintegration times. For the dissolution experiment and the CAF content determination, a phosphate buffer was used with a pH close to that of saliva, which has some advantages, including a higher ionic strength and better buffering capacity. Even if the phosphate buffer presents some advantages compared to water, the ionic strength and viscosity are still different in comparison to those of a simulated saliva fluid or saliva itself. Considering that no pharmacopoeial requirements regarding disintegration behavior have been included in the literature until now, the type of liquid and the method used to evaluate this parameter vary [1,3,5,6,8,10,12,37,38,43,44,47,51,52]. Often, the orodispersible tablets’ recommendation regarding the disintegration time is taken into consideration when establishing this parameter [52].

The liquids that can be used to evaluate the disintegration behavior and the methods that can be selected to evaluate this parameter are highlighted in Table 3 [1,3,5,6,8,10,12,37,38,43,44,47,51,52].

### 3.1. CAF-ODF Preparation

During the study, different polymers were evaluated: polyvinylpyrrolidone, polyvinyl acetate, and AquaPolish^®^ (a mixture of different cellulose esters). In all cases, different issues were observed; for example, the films were sticky or did not become solid after being kept in the oven at 50 °C for 24 h, so the HPMC was the only polymer with which good results were obtained. We considered HPMC-ODF concentrations between 5 and 15% but only concentrations of 8% and 9% led to ODFs that fulfilled the selected critical parameters. Additionally, in some cases, an increased value of the selected film-forming agent produced an extended disintegration time and increased stickiness, whilst a lower concentration produced very low values for the thickness-normalized tensile strength and folding endurance, or the inability to cut the films in a circle shape.

**Figure 3:**
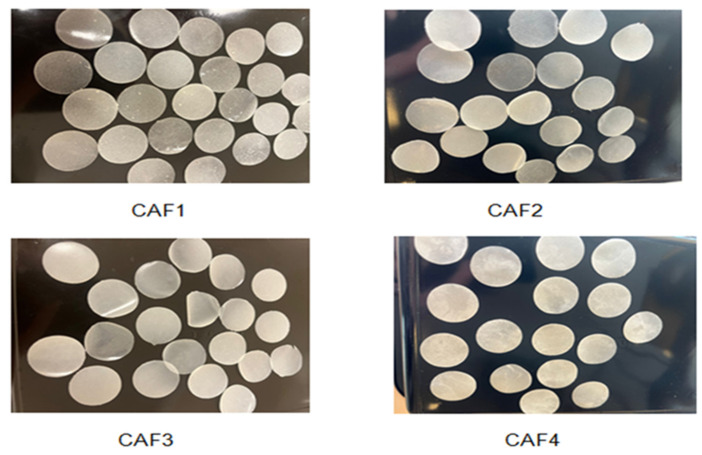
The CAF-ODFs: CAF1 (8% HPMC E 5, CAF), CAF2 (8% HPMC E 5 and CAF:CA–1:1), CAF3 (9% HPMC E 5 and CAF:CA–1:1), and CAF4 (9% HPMC E 5 and CAF:SB–1:1).

Four formulations of CAF-ODFs were prepared with all of them being thin, homogenous, opaque, and slightly white. The part of the film that was in contact with the Petri glass tended to be softer in comparison with the part that was in contact with the environment. CAF-ODFs with a surface area of 3.14 cm^2^ were obtained and were circular-shaped, as can be seen in Figure 3.

### 3.2. The pH of CAF-ODFs

**Figure 4:**
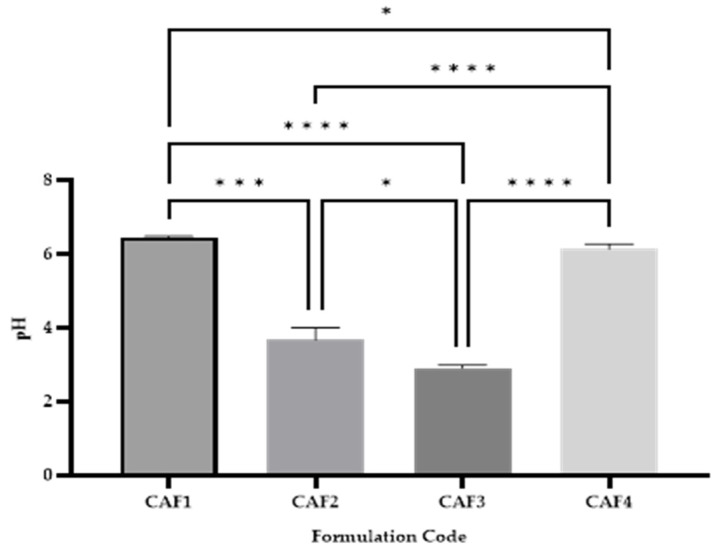
CAF-ODF pH (average ± SD; *n* = 3). CAF1: 8% HPMC E 5, CAF; CAF2: 8% HPMC E 5 and CAF:CA 1:1; CAF3: 9% HPMC E 5 and CAF:CA 1:1; and CAF4: 9% HPMC E 5 and CAF:SB 1:1; * (*p* ≤ 0.05); *** (*p* ≤ 0.001); **** (*p* ≤ 0.0001), Brown-Forsythe and Welch ANOVA test (multiple comparisons); significance level (*p* < 0.05). All unmarked comparisons correspond to non-significant differences (*p* > 0.05).

The surface pH of the buccal cavity ranges between 6.2 and 7.6, and the ODFs should have a pH close to the pre-established values [47]. The determination of pH is very important in order to notice any side effects, due to the fact the acidity or basicity of the film may irritate the mucosal membrane of the oral cavity [47]. The selected hydrotropic substance influences the pH of the CAF-ODFs, as can be seen in Figure 4.

Between all the formulations analyzed (Figure 4), statistically significant differences were recorded: * *p* < 0.05 for CAF1 vs. CAF4 and CAF2 vs. CAF3; *** (*p* < 0.001) for CAF1 vs. CAF3; and **** *p* < 0.0001 in the case of CAF 1 vs. CAF3, CAF2 vs. CAF 4, and CAF3 vs. CAF4, which means that all the variables considered in this study led to statistically significant differences.

The pH (Figure 4) varied from 2.92 ± 0.069 for CAF3 to 6.47 ± 0.02 for CAF1. It can be seen that the formulations CAF1 and CAF4 have a pH close to the one belonging to saliva (6.2–7.6 with interindividual variations), whilst the CAF2 and CAF3 formulations exhibited a more acidic pH, due to the CA used in these two compositions. Even if CAF2 and CAF3 have an acidic pH, these formulations can be used further because CA increases saliva stimulation, leading to a faster disintegration of the film. An impediment for these two formulations might be local irritation, which is lower in the case of CAF1 and CAF4 at the obtained pH.

### 3.3. CAF-ODFs’ Uniformity of Mass

**Figure 5:**
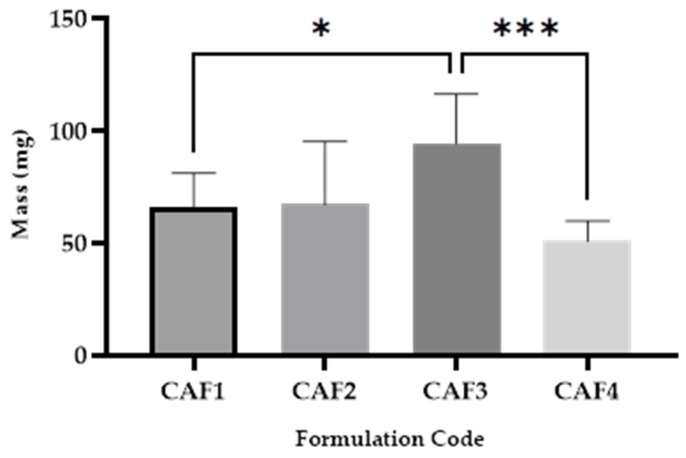
Uniformity of mass of CAF-ODFs (average ± SD; *n* = 20): CAF1: 8% HPMC E 5, CAF); CAF2: 8% HPMC E 5 and CAF:CA 1:1; CAF3: 9% HPMC E 5 and CAF:CA 1:1; and CAF4: 9% HPMC E 5 and CAF:SB 1:1; * (*p* ≤ 0.05); *** (*p* ≤ 0.001); Brown–Forsythe and Welch ANOVA test (multiple comparisons); significance level (*p* < 0.05). All unmarked comparisons correspond to non-significant differences (*p* > 0.05).

For this parameter, the following differences were recorded as significant (Figure 5): CAF1 vs. CAF3 (*), which can be explained by the different amounts of HPMC E 5 (8% for CAF1, 9% for CAF3, and the presence of CA in the case of the third formulation); and CAF3 vs. CAF4, which can be explained by the different hydrotropic substances used: CA for CAF3 and SB for CAF4 (***).

The variation in the average mass can be explained by the amount of HPMC E 5 used (8% for the first two formulations and 9% for the last two formulations). The largest value for the uniformity of mass was registered in the case of CAF3 in which CA and the largest amount of HPMC E 5 were used (Figure 5).

In a study by Drašković et al., CAF was used as the model active ingredient, and the effects of the type of film-forming agent were studied. The formulations were prepared using Kollicoat^®^ (coded as Kx) or Hydroxypropyl cellulose (coded as Hx). The 11th and 12th formulations differed; in the 11th formulation (K11/H11), sodium croscarmellose was used, whilst for the 12th formulation (H12), calcium silicate was used. K12 was excluded from the study due to integrity loss. The researchers obtained values for the uniformity of mass close to the ones presented in this article (between 0.093 ± 0.007 g for H12 and 0.153 ± 0.006 g for K11), highlighting the importance of the film-forming agent and the influence of calcium silicate and sodium croscarmellose on this parameter [43].

In another study was conducted by Sultana et al., in which CAF-ODFs were also developed using the selected film-forming agent HPMC 2910 (with a viscosity of 15 cP) and different amounts for each formulation (1500 mg for F1, 2000 mg for F2, and 2500 mg for F3). The following results were obtained: regarding the uniformity of mass and the disintegration time, both increased in the following order: F3 > F2 > F1, showing the importance of the percentage of the film-forming agent used [44].

### 3.4. CAF-ODF Thickness

Film thickness is an essential parameter because, if it is too thick, the ODF could have a prolonged disintegration and, if it is too thin, it could exhibit poor mechanical properties [52,53].

**Figure 6:**
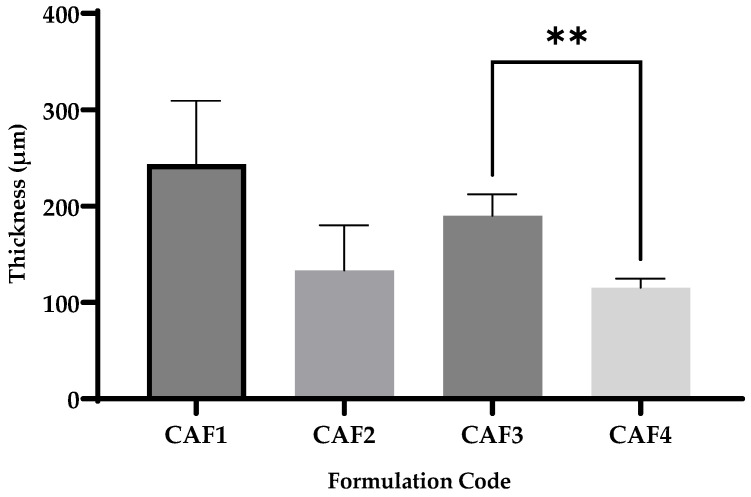
The thickness of CAF-ODFs (average ± SD; *n* = 10). CAF1: 8% HPMC E 5, CAF); CAF2: 8% HPMC E 5 and CAF:CA 1:1; CAF3: 9% HPMC E 5 and CAF:CA 1:1; and CAF4: 9% HPMC E 5 and CAF:SB 1:1; ** (*p* ≤ 0.01), Brown–Forsythe and Welch ANOVA test (multiple comparisons); significance level (*p* < 0.05). All unmarked comparisons correspond to non-significant differences (*p* > 0.05).

The CAF-ODF thickness (Figure 6) varied between 115.4 µm for CAF4 and 243.6 µm for CAF1, for which the largest thickness was registered. For this mechanical parameter, the following statistical difference was observed: CAF3 vs. CAF4 (**), which can be explained through the different hydrotropic substances used (CA for CAF3 and SB for CAF4). For the other formulations, no statistical differences were recorded (Figure 6).

Draskovic et al., developed ODFs with caffeine which presented a film thickness ranging between 146 µm ± 4 µm for H11 and 216 µm ± 10 µm for K11, which are close to the results outlined in this study [43].

In the study conducted by Jain and Mundada, the optimal thickness varied between 50 and 1000 µm, while the results obtained in this study ranged in the previously mentioned interval [54]. In another study that aimed to develop diazepam ODFs, the thickness varied between 243 µm (in which 75 mg of diazepam, 300 mg of HPMC E 3, and 45 mg of polyethylene glycol-PEG_400_ were used) and 632 µm (in which 75 mg of diazepam, 600 mg of HPMC E 15, and 90 mg propylene glycol were used) [55]. Ibrahim et al., developed ODFs with chitosan as a film-forming agent and varying the type and concentration of the plasticizer. In this case, the thickness values varied between 70 µm (in which an acetic acid solution of 2% was used with no plasticizer) and 430 µm (in which a 2% acetic acid solution was used with 10% PEG_400_ as the plasticizer) [56].

### 3.5. CAF-ODFs’ Folding Endurance

**Figure 7:**
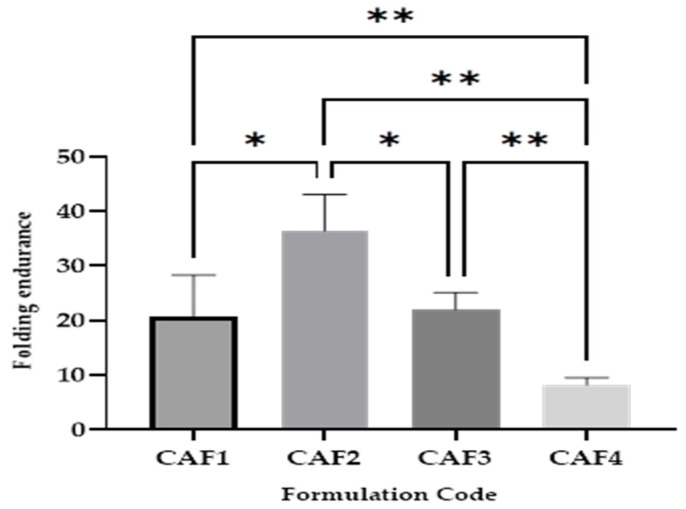
Folding endurance of CAF-ODFs (average ± SD; *n* = 5). CAF1: 8% HPMC E 5, CAF); CAF2: 8% HPMC E 5 and CAF:CA 1:1; CAF3: 9% HPMC E 5 and CAF:CA 1:1; and CAF4: 9% HPMC E 5 and CAF:SB 1:1; * (*p* ≤ 0.05); ** (*p* ≤ 0.01), Brown–Forsythe and Welch ANOVA test (multiple comparisons); significance level (*p* < 0.05). All unmarked comparisons correspond to non-significant differences (*p* > 0.05).

The folding endurance (Figure 7) oscillated between 8 ± 1 folds for CAF4 and 36 ± 6 folds for CAF2. Because in all the formulations, the concentration of the plasticizer was constant, the variation in the folding endurance cannot be attributed to the plasticizer, but it can be correlated with the film-forming agent content. In the case of this parameter, the following differences were revealed: CAF1 vs. CAF2 (*), CAF1 vs. CAF4 (**), CAF2 vs. CAF3 (**), CAF2 vs. CAF4 (**), and CAF3 vs. CAF4 (**). The dissimilarities can be explained through the different amounts of film-forming agent (CAF1 vs. CAF4; CAF2 vs. CAF3; CAF2 vs. CAF4) and also by the type of hydrotropic substance used (CAF1 vs. CAF2, CAF1 vs. CAF4, CAF2 vs. CAF3, CAF2 vs. CAF4, and CAF3 vs. CAF4).

Dwivedy et al., reported that a folding endurance of <25 is suitable for ODFs [57]. In this regard, three of the formulations met this requirement: CAF1, CAF3, and CAF4, whilst CAF2 presented a larger value for the folding endurance. If the folding endurance is >25, but the other properties meet the in-force pharmacopeial requirements or the pre-established minimum or maximum values of the evaluated parameters, it is not considered an impediment.

In the study conducted by Sultana et al., CAF-ODFs were also developed using a film-forming agent with a higher viscosity, HPMC 2910 (a viscosity of 15 cP), in different amounts for each formulation (1500 mg for F1, 2000 mg for F2, and 2500 mg for F3). The following results were obtained for the folding endurance: F3 > F2 > F1, showing the importance of the percentage of film-forming agent used. [44].

### 3.6. CAF-ODFs’ Thickness-Normalized Tensile Strength

**Figure 8:**
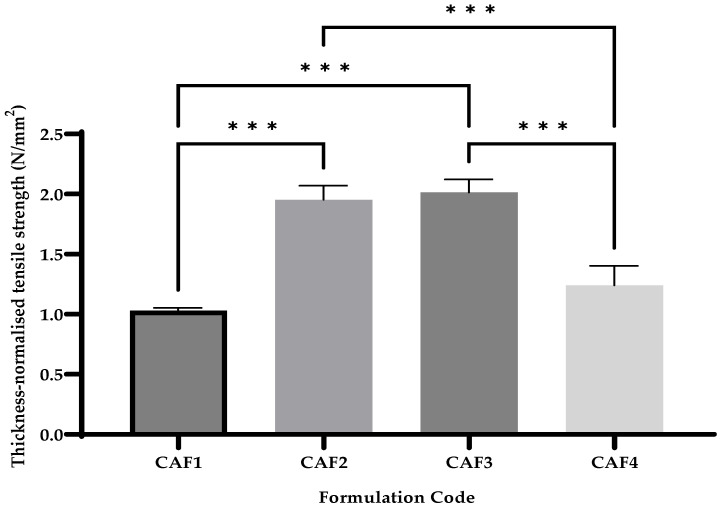
Thickness-normalised tensile strength of CAF-ODFs (average ± SD; *n* = 5). CAF1: 8% HPMC E 5, CAF); CAF2: 8% HPMC E 5 and CAF:CA 1:1; CAF3: 9% HPMC E 5 and CAF:CA 1:1; and CAF4: 9% HPMC E 5 and CAF:SB 1:1; *** (*p* ≤ 0.001); Brown–Forsythe and Welch ANOVA test (multiple comparisons); significance level (*p* < 0.05). All unmarked comparisons correspond to non-significant differences (*p* > 0.05).

Tensile strength is an important parameter regarding the fabrication, manipulation, transportation, and storage of ODFs. In the current study, the thickness-normalized tensile strength ranged between 1.03 ± 0.02 N/mm^2^ (CAF2) and 2.01 ± 0.12 N/mm^2^ (CAF3) (Figure 8). For the thickness-normalized tensile strength, statistical differences were noted in the cases of CAF1 vs. CAF2 (***), CAF1 vs. CAF3 (***), CAF2 vs. CAF4 (***), and CAF3 vs. CAF4 (***); the differences were recorded when using a hydrotropic substance (CA or SB) and when changing the hydrotropic substance.

### 3.7. CAF-ODFs’ Disintegration Time

**Figure 9:**
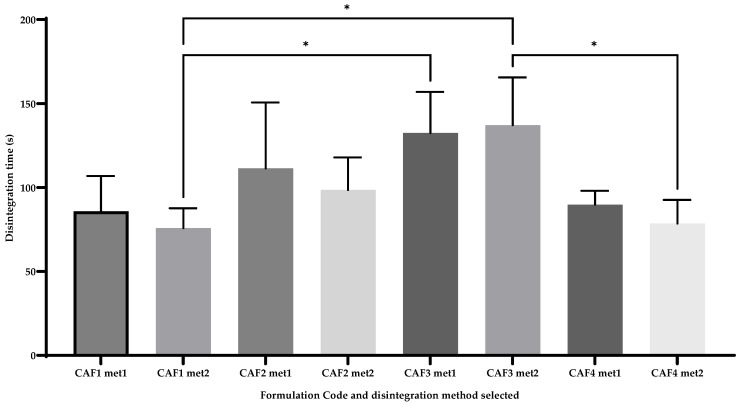
Disintegration times (average ± SD; *n* = 6) measured by the slide frame method (met1) and by the basket-rack method (met2) CAF1: 8% HPMC E 5, CAF); CAF2: 8% HPMC E 5 and CAF:CA 1:1; CAF3: 9% HPMC E 5 and CAF:CA 1:1; and CAF4: 9% HPMC E 5 and CAF:SB 1:1; * (*p* ≤ 0.05), Brown-Forsythe and Welch ANOVA test (multiple comparisons); significance level (*p* < 0.05). All unmarked comparisons correspond to non-significant differences (*p* > 0.05).

The disintegration behavior (Figure 9) was evaluated using two different methods: one which has been described in articles that aim to evaluate ODFs (Method 1 or the slide frame method), while the other is usually employed in laboratories in the pharmaceutical industry (Method 2 or the basket-rack method). Method 1 varied between 85.83 ± 19.16 s for CAF1 and 132.4 ± 21.4 s for CAF3, with similar disintegration times being observed using Method 2 (75.15 ± 10.79 s for CAF1 and 137.16 ± 25.93 s for CAF3). In regards to the European Pharmacopoeia 10th Edition (Ph. Eur. 10) stipulation (Chapter 2.9.1) (with a disintegration time <180 s), all the developed formulations met this requirement [4].

No differences were recorded regarding the first method of disintegration; moreover, the same conclusion can be drawn if the two methods are compared (for the same formulation). Despite this conclusion, some differences were recorded in the case of the second method of disintegration between CAF1 and CAF3 (*), which can be explained by the different amounts of film-forming agent and the use of a hydrotropic substance (CA in the case of CAF3 and no hydrotropic substance in the case of CAF1 and CAF3 and CAF4 (*)).

Draskovic et al., obtained the disintegration times for a CAF-ODF developed that varied between 50 ± 8 s (H12) and 104 ± 19 s (H11), highlighting that if a disintegrant is used in the composition of the ODF, this might lead to an increased disintegration time [43].

In the study conducted by Speer et al., different methods of evaluating the in vitro disintegration time of a prepared ODF were employed. Two of the four methods used in the previously mentioned study were also used in our study. While using the frame method, it was observed that film thickness is a factor that can increase the disintegration time. During the second mentioned method, in which a disintegration test was used, it was noted that all the ODFs disintegrated in less than 60 s [58].

In the study conducted by Khan et al., disintegration times between 40 and 50 s were registered, though the in vivo disintegration time was recorded, whilst in our study, the in vitro disintegration behavior was studied [59]. Usually, the disintegration time varies as a result of the plasticizer used, the type and amount of film-forming agent used, and the presence and absence of APIs. By increasing the amounts of plasticizer and film-forming agent, the disintegration time usually increases; thus, a compromise is needed whilst developing ODFs. The presence or absence of APIs usually influences the disintegration time. By including APIs, this parameter tends to increase, in some cases exceeding the maximum disintegration time limit (180 s) [4]. Both methods can be used whilst evaluating the in vitro disintegration behavior of the CAF-ODFs, but, considering the proposed formulation, method 1 may offer a better correlation with the conditions from the buccal cavity, considering the limited amount of saliva produced.

### 3.8. CAF-ODF Adhesivity

**Figure 10:**
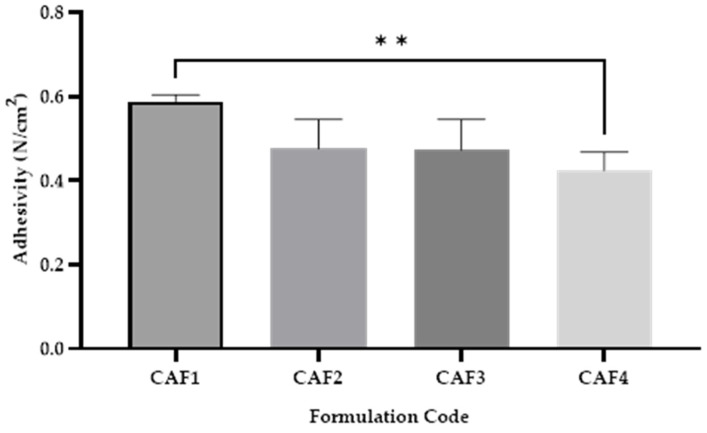
Adhesiveness of CAF-ODFs (average ± SD): CAF1: 8% HPMC E 5, CAF); CAF2: 8% HPMC E 5 and CAF:CA 1:1; CAF3: 9% HPMC E 5 and CAF:CA 1:1; and CAF4: 9% HPMC E 5 and CAF:SB 1:1; ns (*p* > 0.05); ** (*p* ≤ 0.01); Brown–Forsythe and Welch ANOVA test (multiple comparisons); significance level (*p* < 0.05). All unmarked comparisons correspond to non-significant differences (*p* > 0.05).

Adhesivity (Figure 10) is an optional test during the evaluation of ODFs, because ODFs are usually disintegrated quickly in the oral cavity by saliva. However, if a good level of adhesivity is obtained for the ODFs, some disadvantages might be excluded, such as the elimination of the film by swallowing the ODF, which could result in lower efficacy. The results obtained regarding the adhesivity are close for all the formulations, varying between 0.424 ± 0.046 N/cm^2^ (CAF4) and 0.588 ± 0.00 N/cm^2^ (CAF1). The only statistically significant difference (**) was between CAF1 and CAF4, in which the use of SB and the increased value of HPMC E 5 produced a lower amount of adhesivity for CAF4 (Figure 10).

During our manipulations, no issues regarding adhesivity were observed. While being manipulated, the films were not sticky, and during storage, no increased adhesion that might produce fractures in the films was noted.

In the study conducted by Ibrahim et al., CA and acetic acid solutions were used to disperse chitosan. They noted that the films containing CA tended to present better levels of adhesivity in comparison to those of the ones with acetic acid [56].

### 3.9. CAF Content

**Figure 11:**
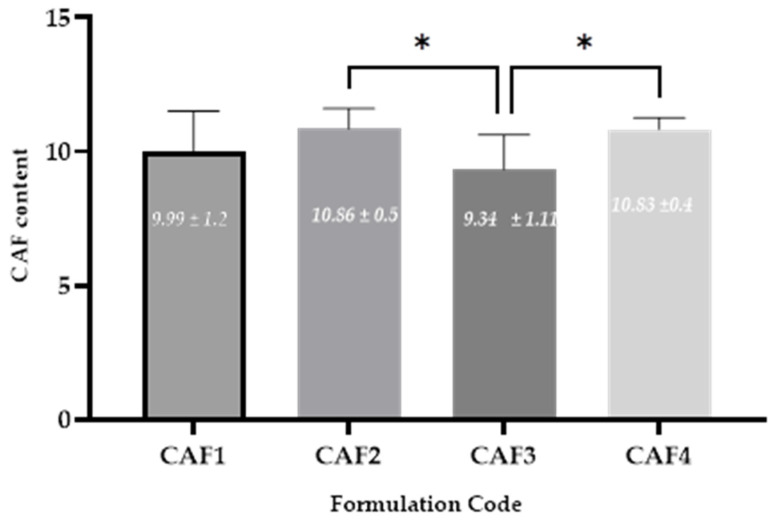
Average CAF-ODF content ± SD: CAF1 (8% HPMC E 5, CAF), CAF2 (8% HPMC E 5 and CAF:CA–1:1), CAF3 (9% HPMC E 5 and CAF:CA–1:1), and CAF4 (9% HPMC E 5 and CAF:SB–1:1), * (*p* ≤ 0.05); Brown-Forsythe and Welch ANOVA test (multiple comparisons); significance level (*p* < 0.05). All unmarked comparisons correspond to non-significant differences (*p* > 0.05).

To evaluate the CAF content from the four ODFs developed, a spectrophotometric UV-Vis method was used. The average CAF content in the formulations varied between 85% and 115% of the average content [4]. Statistically significant differences (Figure 11) were recorded for the following groups: CAF2 vs. CAF3 (the hydrotropic substance was maintained while the HPMC E 5 concentration increased) and CAF3 vs. CAF4 (the film-forming agent concentration was kept constant whilst the hydrotropic substance was changed from CA to SB). The uniformity of the CAF content of the CAF-ODFs met the Ph. Eur. 10 requirements, since each individual content was between 85% and 115% of the average content [4].

Three-dimensional printed CAF orodispersible films were developed by O’Reilly et al., with three different CAF concentrations of 5%, 10%, and 20%. In the case of the lower concentration, the result obtained was close (4.65%) to the pre-established one, whilst for the intermediate concentration and highest concentration of CAF, the results were 7.28% and 15.61% [3].

### 3.10. Dissolution for the CAF-ODFs

**Figure 12:**
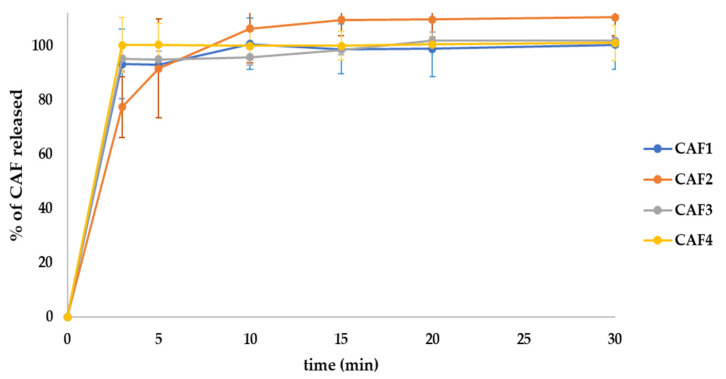
CAF1–CAF4 releasing profiles (average ± SD, *n* = 6): CAF1 (8% HPMC E 5, CAF), CAF2 (8% HPMC E 5 and CAF:CA–1:1), CAF3 (9% HPMC E 5 and CAF:CA–1:1), and CAF4 (9% HPMC E 5 and CAF:SB–1:1).

The dissolution test for the developed CAF-ODFs showed that at 5 min CAF1, CAF2, and CAF3 released over 85% of the APIs, whilst at 30 min all three formulations showed a good dissolution behavior of almost 100% (Figure 12). Regarding the CAF4 formulation, the amount of CAF released at 5 min was 100%. This behavior can be explained by the combination of APIs and the hydrotropic substance (CAF and SB), which presents a higher solubility in hydrophilic dissolution media.

Draskovic et al., used a paddle apparatus, 250 mL of simulated salivary fluid thermostated at 37 ± 0.5 °C, and a wire holder to prevent the films from floating. This research group reported the amounts of CAF released at 15 min were higher than 85%, while in the present study, more than 95% of the APIs were released after 10 min [43]. However, in the study conducted by Sultana et al., (a paddle dissolution test with 250 mL of phosphate buffer with the same temperature and rpm as in our study), over 80% of the CAFs were released after 2 min [44]. The differences regarding the amount of CAF released between this study and these other two studies could be due to the type and volume of dissolution media and the type of agitator used, as the dissolution tends to be lowered in simulated salivary fluid.

## 4. Conclusions

Four slightly white CAF-ODF formulations with a surface area of 3.14 cm^2^ which contained 10 mg of CAF were developed. All four of the formulations proposed exhibited amounts of APIs released that were close to 100% at 30 min, a fact that might imply a fast therapeutic effect. Even if adhesivity is an optional test for ODFs, this study was conducted to prevent the elimination of ODF from one’s mouth or fast swallowing. The mechanical properties were evaluated by the thickness-normalized tensile strength and the folding endurance, both of which showed good results, in accordance with other results from previously published articles. Good mechanical properties are also correlated with no need for special manipulation. The varied composition in the case of the four proposed formulations resulted in differences regarding the evaluated parameters.

All the considered formulation parameters provided differences that were statistically significant considering the pH. Regarding the uniformity of mass, it was observed that by increasing the film-forming agent concentration and by using CA as a hydrotropic substance, a difference from a statistical point of view was noted in comparison from the formulation in which a concentration of 8% HPMC E 5 and no hydrotropic substance were used. The same behavior was noted whilst keeping the higher concentration of the film-forming agent considered in this study constant and by changing the hydrotropic substance (from CA to SB). The latter-mentioned modification regarding the hydrotropic substance also produced a dissimilarity in another evaluated parameter (thickness). The statistical evaluation of the folding endurance showed that by increasing the concentration of HPMC E 5 or by using or changing the hydrotropic substance, statistical differences were recorded. Whilst evaluating the thickness-normalised tensile strength, the only exception in which no significant difference was recorded was in the case of CAF 2 and CAF 3 which used the same hydrotropic substance but the concentration of the film-forming agent was increased (from 8% to 9%). Through the disintegration behavior study, it was observed that only the second method provided a statistical differentiation obtained by increasing the HPMC E 5 concentration and by adding CA, or by keeping the same concentration of the film-forming agent and by replacing the CA with SB. The adhesivity test highlighted that an increased concentration of HPMC E 5 and the use of SB as a hydrotropic substance produced significant differences in comparison with the CAF 1 formulation in which no hydrotropic substance was used and the film-forming agent concentration used was the lowest considered in this study (8%).

## Figures and Tables

**Table 1:** List compositions of CAF-ODFs presented in the literature and marketed CAF-ODFs.

Brand Name/Formulation Code	API	Film-Forming Agent	Reference
CAF-ODFs Registered as Dietary Supplements			
Nanoveda^®^ Energy strips	Caffeine + L-thiamine + Vitamin B12	Pullulan	[39]
Aavishkar oral strips (Caffeine strips)	Caffeine	❖	[40]
BonAyu^®^ Caffeine energy strips	100 mg Caffeine 4 mg Vitamin B6	Maltodextrin, HPC	[41]
Revvies^®^ Energy strips	40 mg Caffeine	Starch, Hydroxypropyl cellulose	[42]
CAF ODF outlined in the literature			
H11/K11/H12	Caffeine	PVA, HPC	[43]
F1–F3	Caffeine	HPMC 2910 (15 cPs)	[44]
F4–F6	Caffeine	Sodium alginate + sodium starch glycolate	[44]
F7–F9	Caffeine	Kollicoat ^®^IR white	[44]
5%, 10%, 15% *w*/*w*	Caffeine	Blanose Carboxymethyl Cellulose Type 7HF-PH	[3]
1% (*w*/*w*)	Caffeine	HPMC/HPMC + PVA	[45]

❖ Manufacturer did not mention the film-forming agent.

**Table 2:** Composition of the studied ODFs.

Ingredient % (*w*/*w*)	Formulation Code			
	CAF1	CAF2	CAF3	CAF4
HPMC E 5	8	8	9	9
1,2-propylene glycol	10	10	10	10
Sucralose	0.2	0.2	0.2	0.2
CAF	2.88	2.88	2.88	2.88
CA	-	2.88	2.88	-
SB	-	-	-	2.88
Ethanol 96% (*v*/*v*)	39.46	38.00	37.50	37.50
Distilled water	39.46	38.00	37.50	37.50
Dispersion weight	100	100	100	100

**Table 3:** The liquids used and the methods employed to establish the disintegration time.

Liquid Used	Method Employed	Reference
Phosphate buffer, pH = 6.8 Phosphate buffer, pH = 6.8 Saliva	Petri dish Drop method In vivo	[1]
Simulated salivary fluid	Oral cavity model	[3]
Phosphate buffer, pH = 6.8	Clamp method	[5]
Simulated salivary fluid	Petri dish method Slide frame method	[6]
Simulated salivary fluid	Clamp method	[8]
Simulated salivary fluid	Clamp method	[10]
Distilled water	Slide frame method Modified pharmacopoeial method	[12]
Deionized water	Petri dish method Drop method	[37]
Simulated saliva	Glass beaker with 20 mL of simulated saliva	[38]
Salivary simulated fluid	Clamp method	[43]
Distilled water	Glass beaker with 25 mL distilled water, swirling every 10 s	[44]
Distilled water	Petri dish method	[47]
Phosphate buffer, pH = 6.75	Clamp method Cell method Frame method Modified USP method Agar plate method	[51]
Water	Pharmacopoeial method	[52]

## Data Availability

Not applicable.
